# Predictors of Poor Quality of Life in Patients with Gastroesophageal Reflux Disease Undergoing Sleeve Gastrectomy

**DOI:** 10.3390/jcm13195825

**Published:** 2024-09-29

**Authors:** Jonathan B. Yuval, Fahim Kanani, Andrei Keidar, Shai Meron Eldar, Eran Nizri, Guy Lahat, Adam Abu-Abeid

**Affiliations:** 1 Division of Surgery, Tel-Aviv Souraksy Medical Center, Tel Aviv 6423906, Israel; kanani.fahim@gmail.com (F.K.); ndrkdr@gmail.com (A.K.); shaime@tlvmc.gov.il (S.M.E.); erann@tlvmc.gov.il (E.N.); guyla@tlvmc.gov.il (G.L.); adama@tlvmc.gov.il (A.A.-A.); 2 Faculty of Medicine, Tel Aviv University, Tel Aviv 6423906, Israel

**Keywords:** sleeve gastrectomy, reflux, quality of life, patient-reported outcomes

## Abstract

**Background**—Gastroesophageal reflux disease (GERD) is commonly diagnosed in patients with severe obesity. The outcomes of patients with preoperative GERD after sleeve gastrectomy (SG) are unclear, and some surgeons consider GERD a contraindication for SG. **Methods**—A retrospective analysis of a tertiary university hospital database was conducted. All patients with preoperative GERD undergoing SG between January 2012 and January 2020 and having at least two years of follow-up were included in the analysis. A validated GERD-associated quality of life questionnaire (GERD-HRQL) was completed by all patients. **Results**—During the study period, 116/1985 patients (5.8%) were diagnosed with GERD before SG. In total, 55 patients were available for a two-year follow-up and were included in the analysis. Median follow-up was 40 months (range 24–156 months). Mean total weight loss (TWL) was 24.0% ± 12.0%. On follow-up, 43 patients (78.1%) reported having GERD symptoms. In patients who underwent postoperative endoscopy, less than a third had esophagitis. The mean GERD-HRQL score was 25.2 ± 10.9. On univariable analysis, patients with poor GERD-HRQL had lower BMI at baseline (41.5 ± 12.4 vs. 44.9 ± 10.0 kg/m^2^, *p* = 0.03), were less commonly smokers at baseline (8.1% vs. 33.3%, *p* = 0.02), and had lower TWL at the end of the follow-up (22.2% ± 10.4% vs. 28.9% ± 13.7%, *p* = 0.05). On multivariable analysis, smoking status at baseline and TWL at last follow-up were independent predictors of better GERD-HRQL. **Conclusions**—In conclusion, most GERD patients after SG have a relatively high GERD-HRQL score, most patients still have GERD symptoms during the follow-up, and approximately a third of patients have endoscopic signs of esophagitis. There was an association between patients with higher TWL and smoking at baseline and better GERD-HRQL outcomes. The latter is potentially due to smoking cessation.

## 1. Introduction

The rate of severe obesity has been continuously increasing since the beginning of the 21st century, and its worldwide prevalence is expected to double by 2035 [1]. Severe obesity is associated with an increased risk of mortality, primarily due to its association with a number of diseases, such as type 2 diabetes, obstructive sleep apnea syndrome, cardiovascular disease, and cancer [2]. Gastroesophageal reflux disease (GERD) is another associated disease, frequently diagnosed in patients with severe obesity. In the obese population, the pathophysiology of GERD is thought to be primarily related to an increase in intra-abdominal pressure rather than a defective esophageal gastric barrier as is the case in patients without severe obesity [3].

Metabolic and Bariatric Surgery (MBS) is the most effective treatment for severe obesity and has been shown to be associated with sustained weight loss, improvement in obesity-related diseases, improvement in quality of life, and increased life expectancy [4]. Sleeve gastrectomy (SG) is the most common MBS worldwide, and according to the latest International Federation for the Surgery of Obesity and Metabolic Diseases (IFSO) survey on MBS trends [5], its adoption rate is continuously increasing. SG has been reported in many studies to be an effective procedure in terms of long-term weight loss and resolution of obesity-associated diseases [6,7,8].

When tailoring the appropriate type of MBS for patients, one of the factors taken into consideration is the presence of GERD. In general, GERD may be considered a relative contraindication for SG as this procedure can exacerbate GERD symptoms due to increased intragastric pressure. Furthermore, nearly a third of patients following SG suffer from GERD at long-term follow-up [7]. Despite that, SG can be considered in patients with GERD under certain conditions, since symptoms may improve due to weight loss and reduction in acid secretion [9].

The purpose of this study is to analyze the outcomes of patients with GERD undergoing SG. The primary endpoint is to assess the quality of life using the GERD health-related quality of life (GERD-HRQL) questionnaire. Secondary endpoints included weight loss, revisional surgery, and endoscopic findings.

## 2. Materials and Methods

### 2.1. Patients

This is a retrospective comparative study based on a prospectively maintained bariatric surgery patient registry in a tertiary, inner city, teaching hospital. The registry was searched between January 2012 and January 2020 for patients with at least two years of follow-up following SG who were also diagnosed preoperatively with GERD. Patients were diagnosed with GERD by clinical symptoms, proton pump inhibitor (PPI) use, or endoscopic or fluoroscopic findings [10]. All patients underwent preoperative endoscopic evaluation of the esophagus, stomach, and duodenum (EGD). All patients were also evaluated by a multidisciplinary team that approved the choice of SG as the preferred MBS procedure. The multidisciplinary team included MBS surgeons, dieticians, social workers, as well as psychologists and/or psychiatrists. When needed, other disciplines were invited to join, most commonly endocrinologists and gastroenterologists. Data captured included baseline demographics: age, sex, body mass index (BMI), previous MBS, smoking at baseline, and obesity-associated medical conditions. Additional data captured included operative and perioperative outcomes, such as complications, revisional surgeries during follow-up, and total weight loss (TWL) at last follow-up. Additionally, all patients completed a validated GERD-associated health-related quality of life questionnaire (GERD-HRQL) at last follow-up [11]. The score of this questionnaire ranges from 0 to 80, with a higher score denoting worse quality of life. Patients with poor GERD-HRQL, defined as a score of 20 or higher, were compared to those with good GERD-HRQL, defined as a score of under 20.

### 2.2. Operative Technique

After signing an informed consent document, patients were brought to the operating theater. Proper identification was made according to the patients’ medical record number and date of birth. A time-out checklist was performed. Patients were placed in supine position. General endotracheal anesthesia was initiated. Patients received 5000 units of subcutaneous heparin and intravenous cefazolin before incision. All procedures were performed in a laparoscopic approach. The greater curvature of the stomach was mobilized approximately 4 cm proximal to the pylorus, and dissected to the angle of His until a clear view of the left crus of the diaphragm was achieved. A 36 Fr Bougie was then inserted along the lesser curvature of the stomach for calibration. Next, the stomach was transected by multiple linear staplers, and the surgical specimen was removed through one of the working ports. There was no routine use of staple-line reinforcement. A routine blue dye leak and patency test was performed.

### 2.3. Postoperative Care

All patients were admitted to the surgical ward following surgery. There, they received high-dose proton pump inhibitors and venous thromboprophylaxis. Patients were instructed to begin ambulation on the day of the surgery. Diet was resumed gradually in the first postoperative day, starting with a liquid diet. Patients were considered eligible for discharge once they tolerated a liquid diet and were ambulating freely.

### 2.4. Follow-Up

Clinical postoperative follow-up occurred at two weeks, four weeks, three months, six months, one year, and then annually. Depending on the individual patient and postoperative course, these intervals were sometimes shortened. During the follow-up, patients were evaluated for weight loss, resolution of obesity-associated medical problems, and chronic MBS complications. Patients were requested to routinely complete blood analyses during follow-up, including micro-nutrient evaluation. In addition, patients were routinely followed by an MBS dietician. Psychosocial follow-up was selectively utilized.

### 2.5. Ethics

The study was approved, and waiver of informed consent was obtained, by our medical center’s institutional review board—approval number TLV-0295-24, received on the 4^th^ of July 2024. The study was conducted according to the ethical standards of the 1964 Declaration of Helsinki and later amendments or comparable ethical standards.

### 2.6. Statistics

Statistical analysis was performed using SPSS statistical software version 29 (IBM SPSS Statistics, Chicago, IL, USA). Categorical data are presented as number (percent) and continuous data are presented as mean ± standard deviation. The chi-square and t-test were used to identify differences between study groups. In multivariable analysis, we used forward method binary logistic regression. The variable selection criterion was a significance of *p*-value < 0.2 in univariable analysis, and conditions for entry and removal were significance 0.05 and 0.1, respectively. For all analyses, a *p*-value ≤ 0.05 was considered statistically significant.

## 3. Results

During the study period, there were 1985 patients who underwent SG. Among these 1985 patients, there were 116 patients (5.8%) diagnosed with GERD preoperatively. Of these 116 patients, 55 patients were available with at least two-year follow-up, and they were included in the study. On preoperative EGD, 52 patients (94.5%) had normal endoscopic findings, 2 patients (3.6%) had grade A esophagitis, and 1 patient (1.8%) had grade B esophagitis.

Baseline characteristics before SG can be seen in Table 1. Mean age and BMI were 43.0 ± 12.0 years and 41.57 ± 5.13 kg/m^2^, respectively. Four patients (7.3%) had a previous laparoscopic adjustable gastric banding (LAGB) before undergoing SG.

Median follow-up was 40 months (range 24–156 months). Mean TWL at the end of follow-up was 24.0% ± 12.0%. At last follow-up, 43 patients (78.1%) reported having GERD symptoms and 45 patients (81.8%) were regularly taking proton pump inhibitor therapy. During follow-up, 34 patients (61.8%) underwent EGD. Of these, 24 patients (70.5% of this subgroup) were found to have normal endoscopic findings, 4 patients (11.8%) had grade A esophagitis, 5 patients (14.7%) had grade B esophagitis, and 1 patient had grade C esophagitis (2.9%). No patients were found to have either grade D esophagitis or Barret’s esophagus on postoperative EGD (Supplementary Table S1). During follow-up, 14 patients (25.5%) underwent surgical revision, mainly due to weight regain. Of the patients who underwent revision, 12 patients (85.7% of this subgroup) underwent conversion to one anastomosis gastric bypass (OAGB), and 2 patients (14.3%) underwent conversion to Roux en Y gastric bypass (RYGB).

The mean GERD-HRQL score was 25.2 ± 10.9. There were 37 patients (67.2%) with poor GERD-HRQL (≥20) and 18 patients (32.7%) with good GERD-HRQL (<20). A comparison of the two groups can be seen in Table 2. On univariable analysis, patients with poor GERD-HRQL had lower BMI at baseline (41.5 ± 12.4 vs. 44.9 ± 10.0 kg/m^2^, *p* = 0.03), were less commonly smokers at baseline (8.1% vs. 33.3%, *p* = 0.02), and had lower TWL at the end of follow-up (22.2% ± 10.4% vs. 28.9% ± 13.7%, *p* = 0.05). There was no significant difference in the length of follow-up between the two groups. In an additional subgroup analysis, there was no significant difference in GERD-HRQL between patients who underwent SG revision to those who did not (24.6 ± 14.0 vs. 25.4 ± 9.9, *p* = 0.83).

In addition to the statistically significant variables in univariable analysis mentioned above (BMI, smoking, and TWL), sleep apnea and postoperative complication of grade 3 or higher were entered into the multivariable analysis since their significance in the univariable analysis was *p* < 0.2 (*p* = 0.13 and *p* = 0.15, respectively). On multivariable analysis, smoking status at baseline and TWL at last follow-up were independent predictors of better GERD-HRQL (*p* = 0.029 and *p* = 0.039, respectively) (Table 2). Smoking at baseline and higher TWL both had a protective effect on the severity of GERD symptoms as captured in the GERD-HRQL questionnaire. In a sensitivity analysis, an additional multivariable logistic regression model including only the three parameters that were statistically significant in univariable analysis showed the same results as the five-parameter model, namely that smoking at baseline and TWL were independently associated with better GERD-HRQL outcomes.

## 4. Discussion

This study evaluated predictors of poor GERD-HRQL in patients with GERD undergoing SG with at least two years of follow-up. We showed that smoking at baseline and high TWL were independent predictors of better GERD-HRQL. High BMI at baseline was associated with better GERD-HRQL at last follow-up on univariable but not multivariable analysis. In addition, we showed that at a median follow-up of 40 months, the majority of patients with GERD undergoing SG have poor GERD-HRQL. In fact, more than two-thirds of patients had a score of 20 or more, with a mean score of 25.2 ± 10.9. Moreover, a significant minority (~30%) of patients who underwent postoperative EGD had signs of reflux esophagitis in follow-up endoscopy.

The underlying pathophysiological causes of GERD are multifactorial. Some causes are more easily addressed than others. For example, the effects of smoking and excess weight/BMI can more easily be reversed by proper counseling and appropriate MBS treatment than motility and functional disorders of the esophagus, lower esophageal sphincter, and stomach. In this study, we showed that those patients with GERD undergoing SG and presenting with or showing improvements in reversible causes of GERD have better GERD-HRQL at last follow-up than those presenting without these reversible factors.

SG, a restrictive MBS, has evolved over the years to become a stand-alone and very common procedure [12]. The popularity of SG could be related to its safety profile when compared to other MBSs [13].

As aforementioned, one of the downsides of SG is the development or aggravation of GERD postoperatively. In a review of the literature by Stenard and colleagues [14], several mechanisms were proposed to cause de novo GERD after SG, and these included decreased gastric compliance, increased intragastric pressure, dysfunction of LES pressure, and abnormal sleeve shapes. Interestingly, they reported mechanisms that could cause GERD resolution following SG, including decreased intra-abdominal pressure due to weight loss, decreased acid secretion due to reduced gastric tissue, and accelerated gastric emptying. The long-term outcomes of SG patients and the rates of GERD during the follow-up have been described by Hauters and colleagues [15], who reported the 10-year outcomes of 34 patients after SG: 22/34 patients (65%) had long-term GERD requiring medical treatment, of which 6/14 (41%) patients had de novo GERD and 16/20 (80%) had persisting GERD. In another retrospective analysis of ten-year outcomes of SG patients, Musella and colleagues [16] reported that 0/4 patients with GERD preoperatively had GERD remission, and 25.7% of the entire cohort were diagnosed with de novo GERD. In a study by Felsenreich and colleagues in which SG patients underwent gastroscopy 15 years following surgery, 55% of patients had symptomatic GERD, and 44% had endoscopic signs of esophagitis [17]. Similarly, in our cohort, a significant minority of patients showed endoscopic signs of esophagitis at a median follow-up of 40 months. Following the analysis of this study and review of the existing literature, we tend to be even more selective when referring GERD patients to SG.

There are a few prospective randomized control trials comparing SG to RYGB that have reported GERD-related outcomes. Salminen and colleagues randomized 240 patients to SG or RYGB with a follow-up of ten years [7] (NCT 00793143). The authors reported better weight loss outcomes in the RYGB group with similar resolution of the associated medical problems between groups. However, the rate of esophagitis in patients after SG was significantly higher than in patients after RYGB (31% vs. 7%, *p* < 0.001). Peterli and colleagues randomized 217 patients to SG or RYGB with a follow-up of five years [8] (NCT 00356213). The primary outcome was excess BMI loss, which was not significantly different between the study groups at five years. Regarding GERD, the SG group had less remission of GERD and a higher proportion of patients with worsening or de novo development of GERD than the RYGB group. Interestingly, the proportion of patients with improvement in GERD or stable GERD was not significantly different between the study groups, perhaps due to insufficient statistical power to detect modest differences in small patient subgroups. Biter and colleagues recently published the SleeveBypass trial in which 628 patients were randomized to SG or RYGB [18] (Dutch Trial Register NTR 4741). At a follow-up of five years, weight loss outcomes were better in the RYGB study group. De novo GERD was more common in the SG study group (16% vs. 4%, *p* < 0.001). De novo GERD may have been chosen as an outcome in the study because at baseline the rate of GERD was higher in the RYGB group in comparison to the SG group (11% vs. 8%, *p* < 0.001). It is interesting to note that during the preparation of this manuscript, there were no published randomized control trials comparing SG to other common MBS procedures, such as OAGB, single anastomosis duodeno-ileal bypass with sleeve gastrectomy (SADI-S), duodenal switch (DS), and others.

Quality of life after SG has been reported throughout the years with some controversies in the scientific literature. Felsenreich and colleagues [19] evaluated quality of life in patients ten years after SG using questionnaires. They concluded that excess weight loss <50% and symptomatic reflux are the main impairments to patients’ quality of life. Fiorani and colleagues [20] showed that SG patients had a poorer quality of life at 8 years when compared to RYGB patients. However, in a retrospective cohort study reported by Alqahtani and colleagues [21], 83% of patients reported that their quality of life after SG was good to excellent. In our cohort, GERD-HRQL score after SG was shown to be better in patients with higher TWL. This is possibly related to the anti-reflux effects of weight loss. In this study, smokers at baseline also showed better GERD-HRQL than non-smokers. At our center, all MBS patients are counseled to quit smoking prior to surgery and encouraged to avoid smoking for the rest of their lifetime. Although we do not have an accurate assessment of the rate of smoking cessation, it is our assumption that most patients do quit smoking prior to MBS. Cessation of smoking could have been an additional contributing factor to improved GERD-HRQL.

GERD resolution and/or improvement after SG is another controversial topic. On the one hand, and similar to the randomized control trials described above, in a national analysis by Dupree and colleagues [22], 84.1% of GERD patients undergoing SG still had GERD symptoms during follow-up, which was higher than 19.8% of GERD patients undergoing RYGB (*p* < 0.05). Interestingly, the authors also reported that patients with preoperative GERD had lower weight loss following SG than patients without GERD. Conversely, in a prospective observational study by Rebecchi and colleagues [23], the authors evaluated gastroesophageal function following SG in 71 patients by questionnaire, endoscopy, pH-monitoring, and manometry. In those patients with preoperative GERD, SG improved symptoms and controlled reflux in the majority of cases without affecting lower esophageal pressure or esophageal peristalsis. Likewise, in a commentary by Gagner [9], he emphasizes that the “hysteria” on GERD or even esophageal adenocarcinoma after SG has to be tempered. Ganger uses clinical, endoscopic, pH-metric, and manometric evidence to state that in most patients with preoperative GERD, SG improves symptoms and reduces reflux [9]. He also notes that SG could be utilized in 99% of patients with severe obesity. In our study, we showed that most (around 80%) still had GERD symptoms and/or were still consuming PPI. However, in postoperative endoscopy, less than a third of patients had any form of esophagitis, and none of the patients had grade D esophagitis or dysplastic changes. We believe that the different outcomes in the literature are probably related to differences in the definition of GERD resolution, which varies in published studies. We recommend individualizing SG for patients with GERD after a multidisciplinary counseling and careful patient selection.

This study has some limitations. It is retrospective in nature and subject to biases related to this design. In addition, the study comprises a relatively small number of patients, with no control group. The proportion of patients available for endoscopic follow-up was relatively low. It is important to note that patients included in the study had mild GERD symptoms and had either mild or no findings on preoperative endoscopy. We refer patients with GERD for SG very selectively; in cases of severe clinical and endoscopic reflux, we generally perform gastric bypass, which explains the low number of pathologic endoscopic findings in this population. Also, the study was conducted at a single tertiary, inner city, teaching hospital. All these factors may limit generalizability. In the cohort, four patients underwent LAGB prior to SG. LAGB is known to cause motility disorders and reflux-associated symptoms, and the choice of SG and their postoperative outcome could be questioned. We wanted to include a real-world cohort of patients at our center undergoing SG with GERD, which included some LAGB patients. We believe including these patients makes our findings more generalizable.

Despite these limitations, the current study has several strengths, which include the relatively long duration of follow-up, the uniformity of performing all surgeries and follow-up at a single center, and the high completion rate of the GERD-HRQL questionnaire. Further studies including prospective randomized controlled trials with a large number of patients and including additional common MBS procedures (e.g., OAGB, SADI) could help discern the most appropriate MBS for GERD patients and may also help define the role of SG for these patients.

## 5. Conclusions

In conclusion, most GERD patients after SG have a relatively poor GERD health-related quality of life, and most patients still have clinical GERD during follow-up. In addition, in approximately one-third of patients, there were endoscopic signs of esophagitis. There was an observed association between patients with increased TWL and smoking at baseline and better GERD-HRQL outcomes, most probably due to decreased intra-abdominal pressure in the former, and due to the ameliorating effects of smoking cessation in the latter. SG could be considered selectively in some patients with GERD, particularly those patients with a higher weight-loss potential, since intra-abdominal pressure may be a main contributor to pathophysiology.

## Figures and Tables

**Table 1:** Baseline characteristics of patients before sleeve gastrectomy.

Characteristic	N = 55 Number (%) or Mean ± Standard Deviation
Female Sex	39 (70.9)
Age (years)	43.0 ± 12.0
Baseline BMI (kg/m^2^)	41.57 ± 5.13
Previous LAGB	4 (7.3)
Smoking	9 (16.4)
Type 2 Diabetes	13 (23.6)
Hypertension	22 (40.0)
Hyperlipidemia	23 (41.8)
Sleep Apnea	9 (16.4)
Osteoarthritis	6 (10.9)
Shortness of Breath	2 (3.7)
Fatty Liver	44 (80.0)

BMI, body mass index; LAGB, laparoscopic adjustable gastric banding.

**Table 2:** Univariable and multivariable analysis of characteristics associated with poor gastroesophageal-reflux-disease-related quality of life.

Characteristic	HRQL ≥ 20 N = 37	HRQL < 20 N = 18	*p*-Value Univariable	*p*-Value Multivariable
	Number (%) or Mean ± Standard Deviation			
Female Sex	26 (70.3)	13 (72.2)	0.88	
Age (years)	41.5 ± 12.4	44.9 ± 10.0	0.32	
Baseline BMI (kg/m^2^)	40.6 ± 4.1	43.4 ± 6.4	0.03	0.16
Previous LAGB	3 (8.1)	1 (5.6)	0.73	
Smoking	3 (8.1)	6 (33.3)	0.02	0.03
Type 2 Diabetes	9 (24.3)	4 (22.2)	0.86	
Hypertension	13 (35.1)	9 (50.0)	0.29	
Hyperlipidemia	15 (40.5)	8 (44.4)	0.78	
Sleep Apnea	8 (21.6)	1 (5.6)	0.13	0.43
Osteoarthritis	3 (8.1)	3 (16.7)	0.34	
Shortness of Breath	2 (5.6)	0 (0.0)	0.31	
Fatty Liver	29 (78.4)	15 (83.3)	0.67	
Severe Complication	4 (10.8)	0 (0.0)	0.15	0.29
Follow-Up (months)	58.8 ± 40.4	47.6 ± 28.1	0.30	
%TWL	22.2 ± 10.4	28.9 ± 13.7	0.05	0.04

HRQL, health-related quality of life; BMI, body mass index; LAGB, laparoscopic adjustable gastric banding; %TWL, percent total weight loss.

## Data Availability

The authors are willing to make the data of this study available upon request.

## Supplementary Materials

The following supporting information can be downloaded at: https://www.mdpi.com/article/10.3390/jcm13195825/s1, Table S1: Endoscopic Findings at Baseline and During Follow Up.
