# Supplementary material for: Predictors of Poor Quality of Life in Patients with Gastroesophageal Reflux Disease Undergoing Sleeve Gastrectomy
Source: J Clin Med. 2024 Sep 29;13(19):5825. doi: 10.3390/jcm13195825 (PMC11478163; doi:10.3390/jcm13195825)
Supplement: Supplementary file 1 [file jcm-13-05825-s001.zip › jcm-3205682-supplementary.pdf]

Supplementary Table S1. Endoscopic Findings at Baseline and During Follow Up.

	**Baseline Endoscopy**<br><br>**N= 55**<br><br>**Number (%)**	**Follow Up Endoscopy**<br><br>**N= 34**<br><br>**Number (%)**
**Normal Findings**	52 (94.5)	24 (70.5)
**Grade A Esophagitis**	2 (3.6%)	4 (11.8)
**Grade B Esophagitis**	0	5 (14.7)
**Grade C Esophagitis**	1 (1.8%)	1(2.9)
**Grade D Esophagitis**	0	0
**Barret's Esophagus**	0	0
